# Supraspinatus tendon thickness and subacromial impingement characteristics in younger and older adults

**DOI:** 10.1186/s12891-022-05179-y

**Published:** 2022-03-11

**Authors:** Tomonobu Ishigaki, Koichiro Yoshino, Motoki Hirokawa, Makoto Sugawara, Masanori Yamanaka

**Affiliations:** 1 Graduate School of Human Life Design, Toyo University, Oka 48-1, Asaka, Saitama, 351-8510 Japan; 2 Graduate School of Health Science, Hokkaido University, Sapporo, Hokkaido Japan; 3 Matsuda Orthopedic Memorial Hospital, Sapporo, Hokkaido Japan; 4 Obihiro Kosei Hospital, Obihiro, Hokkaido Japan; 5 Department of Physical Therapy, Chitose Rehabilitation College, Chitose, Hokkaido Japan

**Keywords:** Acromiohumeral distance, Extrinsic factor, Intrinsic factor, Occupation ratio, Supraspinatus, Tendon abnormalities

## Abstract

**Background:**

Subacromial impingement (SAI) may be a cause of age-related rotator cuff abnormalities; therefore, the purpose of this study was to compare SAI characteristics between younger and older adults. In addition to the fact that thickened supraspinatus tendon (SST) indicates tendon abnormalities, SAI characteristics have been recognized as follows: greater SST thickness, reduced acromiohumeral distance (AHD), greater reduction of AHD (∆AHD) with arm elevation, and a higher percentage of SST within AHD (i.e., occupation ratio: OcAHD). Furthermore, we investigated the relationships between SST thickness and AHD, as well as SST thickness and ∆AHD to clarify the effect of SAI on rotator cuff abnormalities.

**Methods:**

Healthy younger (*n* = 18, 21–24-year-old) and older (*n* = 27, 45–80-year-old) adults without any shoulder symptoms participated in this study. We measured their SST thickness and AHD at rest and at arm elevation (30° and 60°) in the scapular plane using ultrasound, and calculated ∆AHD as the relative change expressed as a percentage of the baseline. OcAHD was expressed as the ratio of SST thickness at rest to AHD at rest and in elevated positions.

**Results:**

The older subjects had approximately one mm thicker SST (*P* = 0.003, 95% Confidence interval [CI] = 0.410 to 1.895) and approximately 1.0 to 1.3 mm greater AHD than the younger subjects (*P* = 0.011, 95%CI = 0.284 to 2.068 at rest; *P* = 0.037, 95%CI = 0.082 to 2.609 for 30° of arm elevation; *P* = 0.032, 95%CI = 0.120 to 2.458 for 60° of arm elevation). However, there were no differences in ΔAHD and OcAHD between the groups.

**Conclusion:**

This study demonstrated that, compared with the younger subjects, the older subjects showed thicker supraspinatus tendon but no other SAI characteristics including decreases in AHD and increases in OcAHD. Thus, this study suggests that older subjects showed age-related SST abnormalities without SAI, although the magnitude of the differences in SST thickness is notably small and the clinical significance of this difference is unclear.

## Background

Aging has been recognized as a risk factor for rotator cuff tears [1–5]. For example, Yamamoto et al. reported that the prevalence of rotator cuff tear in general population was 25.6% in their 60s, 45.8% in their 70s, and 50.0% in their 80s [5]. Degenerative histopathological changes in rotator cuff tendons exist before the tear [6], hence the rotator cuff tear in older individuals may result from age-related rotator cuff abnormalities. Therefore, it is worth investigating the underlying mechanisms for the age-related rotator cuff abnormalities to establish preventive approaches for degenerative rotator cuff tears.

Tendon thickening seems to be associated with rotator cuff abnormalities [7–9]. It is known that patients with various tendinopathy show thicker tendons [10–14]. Moreover, several studies measured rotator cuff tendon thickness using ultrasound (US) and reported that patients with subacromial impingement (SAI) syndrome and rotator cuff tendinopathy showed thickened rotator cuff tendons [7, 8, 15]. In addition, our previous study reported that, even in healthy college baseball players, increases in supraspinatus tendon thickness are associated with greater glenohumeral internal rotation deficits, which have been recognized as a risk factor for throwing-related shoulder injuries [16]. These studies [7–12, 16] indicate that increases in tendon thickness are associated with tendon abnormalities. Further, older individuals also had thickened supraspinatus tendons, that are most frequently involved in rotator cuff tear [17], compared with healthy younger individuals [9]. Considering the results of this study and the fact that the prevalence of rotator cuff tears increases with age [1–5], increases in supraspinatus tendon thickness in older individuals imply age-related supraspinatus tendon abnormalities. However, the underlying mechanism for the increase in supraspinatus tendon thickness in older individuals remains unclear.

SAI is a result of mechanical compression of the rotator cuff tendon in the reduced subacromial space [18]. Although the relationship between SAI and rotator cuff tendon injuries is still controversial, SAI has long seemed to be the major extrinsic cause of rotator cuff tendinopathy [19, 20]. Patients with SAI presented specific characteristics in comparison with healthy controls, including decreased subacromial space [21] and greater reduction in subacromial space during arm elevation [22, 23]. Furthermore, those patients also showed greater occupation ratio (OcAHD) [7], that is the percentage of the supraspinatus tendon (SST) thickness within the subacromial space [7, 24]. The acromiohumeral distance (AHD), which is measured by US, has been used to evaluate the subacromial space [7, 25, 26]. To evaluate reduction in AHD, the changes in AHD (∆AHD) were calculated as the differences in AHD among different arm positions [22, 23, 27]. Besides that the SAI is believed to be the pathomechanism of rotator cuff abnormalities, older people showed similar scapular kinematics alterations to patients with SAI [28]; thus, there may be a relationship between SAI and age-related SST abnormalities. However, the effect of aging on SAI characteristics (i.e., smaller AHD, greater reduction in ∆AHD, and greater OcAHD) is poorly documented. If age-related SST abnormalities potentially develop with SAI, the SAI characteristics would be apparent in older individuals compared to younger individuals.

This study aimed to compare SST thickness and the following SAI characteristics between younger and older individuals: AHD, ∆AHD, and OcAHD. We hypothesized that older individuals would display thicker SST and SAI characteristics when compared with younger individuals. Furthermore, because SAI characteristics were thought to affect SST thickness, we investigated whether SST thickness relates to AHD, and SST thickness to ∆AHD with arm elevation. The results of the present study provide insights into the pathogenesis of rotator cuff abnormalities with advancing age.

## Methods

### Subjects

Eighteen younger adults (10 male and 8 female) and 27 older adults (7 male and 22 female) participated in this study (Table 1). Because a previous study found asymptomatic rotator cuff tears in people in their 40s [29], inclusion criteria for the older group included 40-year-olds or older. In contrast, no rotator cuff tears were found in people in their 20s [5]; thus, the inclusion criteria for the younger adults was people in their 20s. Exclusion criteria for both groups were: individuals with a history of orthopedic disease affecting the shoulder and spine: those with a history of neurological, metabolic, and cardiovascular diseases: and those presenting with shoulder pain at the time of measurement. We explained the purpose of this study and the procedures to be carried out to the subjects, and written informed consent was obtained from all participants before examination. The Institutional Review Board of Hokkaido University approved this study, and all procedures were carried out in accordance with relevant guidelines and regulations.

**Table 1:** Anthropometrics data (mean ± standard deviation [SD])

	Younger	Older
Age (year-old)	22.4 ± 0.8	63.0 ± 7.7*^a^
Height (cm)	167.2 ± 9.9	157.8 ± 9.2*^b^
Body mass (kg)	58.9 ± 8.2	60.4 ± 12.8*^c^

*: Significant difference between groups

^a^ *P* < 0.001; *t* (26.808) = 27.206; 95% confidence interval [CI] = 36.875 to 44.236

^b^ *P* = 0.002; *t* = − 3.282; 95%CI = − 15.218 to − 3.634

^c^ *P* = 0.632; *t* = 0.482; 95%CI = − 4.810 to 7.828

### US measurement

For US imaging, MyLab 25 (Biosound Esaote, Indianapolis, IN, USA) and FAZONE CB (FUJIFILM Co., Tokyo, Japan) with 10–12 MHz linear array transducers in gray scale B-mode were used for the younger and older subjects, respectively. We adjusted imaging parameters to obtain clear images in each subject. We used a built-in caliper system in each US unit to perform digital measurements of the SST thickness and AHD. A single examiner (T.I.) with more than 3 years of experience using the US in orthopedic clinical and research fields acquired all images from the shoulder of the subjects’ dominant arm.

**Fig. 1:**
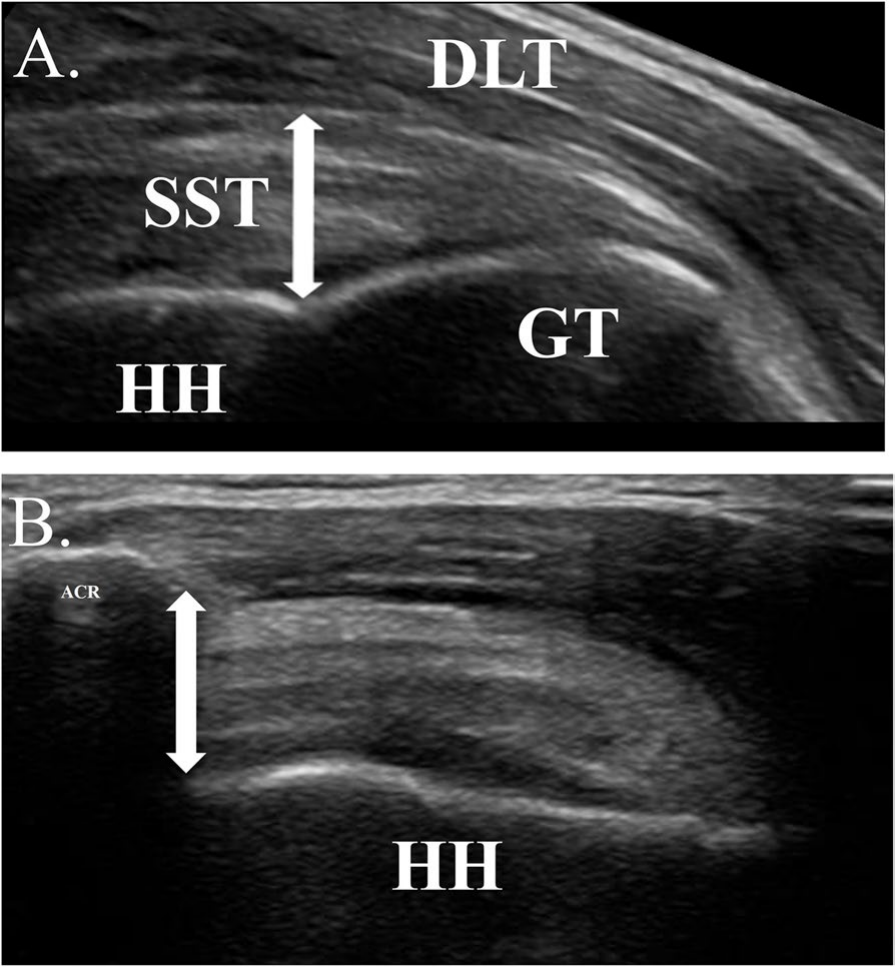
Ultrasound measurement for **a**) supraspinatus tendon thickness (double-ended arrow), and **b**) acromiohumeral distance (double-ended arrow). SST: Supraspinatus tendon, DLT: Deltoid muscle, HH: Humeral head, GT: Greater tuberosity, ACR: Acromion

For the SST measure, the participants were seated in an upright position with their feet flat on the floor. They were asked to place their tested hand on the ipsilateral iliac crest with the elbow pointed posteriorly (the modified Crass position) [24, 30, 31]. We placed the US transducer on the anterior aspect of the acromion in the coronal plane at the position where the footprint at the greater tuberosity and superior facet of the greater tuberosity were visualized. We obtained two longitudinal tendon images at this point and measured SST thickness as the distance between the deep fibers of the tendon and the peribursal fat perpendicular to the tendon fibers at the footprint-cartilage junction on the humeral head (Fig. 1a) in each image [32]. The average of two images for each measurement was used for statistical analysis. In our preliminary study with 14 healthy young individuals, the same examiner performed the described measurement procedure on two separate days to evaluate intra-rater repeatability using the intraclass correlation coefficient (ICC _(1,2)_); consequently, the high repeatability of this measurement procedure was confirmed (ICC _(1,2)_ = 0.94).

US images of AHD were captured with participants seated on a chair in an upright posture, elbows straight, and feet flat on the floor. We placed the transducer parallel to the flat superior aspect of the acromion at the middle portion of the acromion in the plane of the scapula. US images were obtained at three arm angles: at rest (AHD0) with the arm by their side, at 30° (AHD30), and at 60° (AHD60) actively elevated on the scapular plane. For the measurement at an elevated position, the participants actively elevated their arms until the desired angle on the scapular plane with a 1 kg dumbbell in their dominant hands, and were asked to hold the position while the US image was captured. Second examiners (K.Y. or M.H.) confirmed the desired angles with a goniometer and visually confirmed the plane of elevation. This was because an applied external load caused an apparent change in AHD during arm elevation [33]. The elevation angle was determined using a digital inclinometer. The order of the measurement positions was randomized to prevent the effect of fatigue. Participants were also well rested between trials. We acquired two clear images that visualized the acromion and humeral head in each examined position, and measured AHD as the shortest linear distance between the inferior edge of the acromion and the superior aspect of the humeral head (Fig. 1b). For each arm angle, the average AHD from two images was used for further analysis. In our preliminary study on 15 healthy young individuals, the test-retest reliability of the described procedure was good to excellent (ICCs _(1,2)_ for AHD0, AHD30, and AHD60 were 0.87, 0.75, and 0.93, respectively).

The rate of changes in AHD (∆AHD) were calculated as the relative change expressed in the percentage of AHD0 (∆AHD0–30 and ∆AHD0–60) using the following equation.

$$\mathrm{AHD}0-30\ \left(\mathrm{or}\ 0-60\right)\left(\%\right)=\left[\mathrm{AHD}0-\mathrm{AHD}30\ \left(\mathrm{or}\ 60\right)\right]\cdot {\left(\mathrm{AHD}0\right)}^{-1}\cdot 100$$

Corresponding to previous studies [7, 24], the occupation ratio (OcAHD) was expressed as the ratio of the SST thickness to AHD at each arm position (OcAHD0, 30, and 60) using the following equation:

$$\mathrm{OcAHD}\left(\%\right)=\mathrm{SST}\cdot {\mathrm{AHD}}^{-1}\cdot 100$$

### Statistical analysis

IBM SPSS ver. 18 (IBM Corp., Armonk, NY, USA) was used for the statistical analysis. An alpha level was set at 0.05 to determine statistical significance. All descriptive statistics were reported as the mean and standard deviation (SD). Before statistical analysis, we performed the Shapiro-Wilk test to check the normality of SST thickness. Depending on the results of normality, an independent t-test or a Mann-Whitney U test was used to compare SST thickness between young and older subjects. Additionally, we conducted power analysis for SST thickness using G*Power (University of Kiel, Germany). For AHD, ∆AHD, and OcAHD, two-way repeated-measures analysis of variance (ANOVA) was performed to test the effects of age and angle. If a significant interaction effect and main effects were identified, a post-hoc test was conducted with the Bonferroni correction for multiple pairwise comparisons. The effect size was reported using partial eta squared, with small, medium, and large effects classified as 0.01, 0.06, and 0.14, respectively [34]. Furthermore, relationships between SST thickness and AHD, along with ∆AHD, were examined with Pearson’s correlation coefficients in the three different categories: 1) the younger subjects, 2) the older subjects, and 3) all subjects.

## Results

Table 2 summarizes all the descriptive data. Due to the normal distribution of SST thickness, we performed an independent *t*-test to compare group differences. Consequently, SST was approximately 1 mm thicker in the older subjects than their younger counterparts (*P* = 0.008; *t* = 3.135; 95% confidence interval [CI] = 0.410 to 1.900). The power analysis showed 84% power for the detection of differences in SST thickness. For AHD, a two-way repeated ANOVA showed significant main effects of group (*d.f.* = 1.0; *F* = 7.440; *P* = 0.009; p*η*^2^ = 0.148) and angle (*d.f*. = 1.706; *F* =; 94.478; *P* < 0.001; p*η*^2^ = 0.687) but no significant group-by-angle interaction (*d.f*. = 1.706; *F* = 0.054; *P* = 0.925; p*η*^2^ = 0.001). Post hoc analysis revealed that the older subjects had greater AHD than the younger subjects at all elevation angles (Table 2). There was also a significant reduction in AHD between baseline and both elevated positions in both age groups (Table 2), but no significant difference was found between AHD30 and AHD60 (*P* = 1.000; 95%CI = − 0.601 to 1.164 for the older subjects, *P* = 1.000; 95%CI = − 0.856 to 1.306 for the younger subjects). For ∆AHD, there were no significant main effects of interaction (*d.f.* = 1.0; *F* = 0.114; *P* = 0.214; p*η*^2^ = 0.003 for effect of group, *d.f.* = 1.0; *F* = 0.818; *P* = 0.371; p*η*^2^ = 0.019 for effect of angle, and *d.f*. = 1.0; *F* = 0.010; *P* = 0.920; p*η*^2^ = 0.000 for interaction,). For OcAHD, there was a significant main effect of angle (*d.f*. = 2.0; F = 59.576; *P* < 0.001; p*η*^2^ = 0.581), whereas no significant main effect of group (*d.f.* = 1.0; *F* = 0.571; *P* = 0.454; p*η*^2^ = 0.013) or interaction (*d.f.* = 2.0; *F* = 0.056; *P* = 0.946; p*η*^2^ = 0.001) was found. According to the post hoc analysis, OcAHD30 and OcAHD60 were significantly greater than in OcAHD0 (Table 2). However, there was no significant difference between OcAHD30 and OcAHD60 (*P* = 1.000; 95%CI = − 10.388 to 5.838).

**Table 2:** Descriptive data (mean ± standard deviation [SD])

	Younger	Older	Mean difference (95%CI)
SST thickness (mm)	5.2 ± 0.9	6.3 ± 1.6^*a^	1.153 (0.410 to 1.900)
AHD0 (mm)	9.6 ± 1.0	10.8 ± 1.7^*b^	1.176 (0.284 to 2.068)
AHD30 (mm)	6.6 ± 1.5^†e^	7.9 ± 2.4^*c, †g^	1.345 (0.082 to 2.609)
AHD60 (mm)	6.4 ± 1.4^†f^	7.6 ± 2.2^*d, †h^	1.289 (0.120 to 2.458)
ΔAHD0–30 (%)	31.6 ± 14.0	26.5 ± 19.6	−5.136 (−16.162 to 5.890)
ΔAHD0–60 (%)	34.3 ± 10.2	29.8 ± 13.6	−4.445 (− 12.184 to 3.295)
OcAHD0 (%)	53.9 ± 9.0	59.6 ± 16.2	5.648 (− 2.820 to 14.115)
OcAHD30 (%)	82.0 ± 22.3^†i^	86.0 ± 28.5^†i^	4.010 (− 12.090 to 20.111)
OcAHD60 (%)	84.3 ± 20.9^†j^	88.2 ± 28.3^†j^	3.817 (− 11.905 to 19.538)

*CI* Confidence interval, *SST* Supraspinatus tendon, *AHD* Acromiohumeral distance, *ΔAHD* rate of change in acromiohumeral distance, *OcAHD* Occupation ratio

^*^: Significant difference between groups

^†^: Significant difference from baseline (i.e. at rest)

^a^ *P* = 0.008; *t* = 3.135

^b^ *P* = 0.011

^c^ *P* = 0.037

^d^ *P* = 0.032

^e^ *P* < 0.001; 95%CI = 1.930 to 4.198

^f^ *P* < 0.001; 95%CI = 2.513 to 4.064

^g^ *P* < 0.001; 95%CI = 1.968 to 3.821

^h^ *P* < 0.001; 95%CI = 2.543 to 3.809

^i^ *P* < 0.001; 95%CI = − 35.089 to − 19.378

^j^ *P* < 0.001; 95%CI = − 35.911 to − 23.106

The results of Pearson’s correlation coefficients are shown in Table 3. There was no relationship between SST thickness and AHD, as well as ∆AHD, in the younger, the older, and all subjects.

**Table 3:** The relationships between supraspinatus tendon thickness and each variable

	Younger	Older	All participants
AHD0	*r* = 0.290, *P* = 0.243	*r* = 0.061, *P* = 0.763	*r* = 0.234, *P* = 0.122
AHD30	*r* = 0.079, *P* = 0.756	*r* = 0.044, *P* = 0.826	*r* = 0.167, *P* = 0.274
AHD60	*r* = 0.030, *P* = 0.907	*r* = 0.029, *P* = 0.887	*r* = 0.151, *P* = 0.321
ΔAHD0–30	*r* = 0.054, *P* = 0.832	*r* = − 0.048, *P* = 0.813	*r* = − 0.080, *P* = 0.601
ΔAHD0–60	*r* = 0.169, *P* = 0.502	*r* = − 0.029, *P* = 0.885	*r* = − 0.057, *P* = 0.712

*AHD* Acromiohumeral distance, *ΔAHD* rate of change in acromiohumeral distance

## Discussion

There have long been controversies on the mechanism underlying age-related rotator cuff abnormalities. Because SAI is a possible extrinsic mechanism of rotator cuff abnormalities in older individuals, this study compared SAI characteristics between healthy younger and older subjects. This comparison showed that the older subjects had supraspinatus tendons that were significantly thicker than those of the younger subjects. This result supported our hypothesis; contrary to our expectations, the older subjects had greater AHD than the younger subjects, and no significant differences were found in ∆AHD and OcAHD between the two groups across all three arm positions.

Consistent with a previous study [9], this study demonstrated that SST was thicker even in healthy older subjects with no prior shoulder injuries than younger adults. Such thickened tendons have been observed in patients with various types of tendinopathies involving the rotator cuff, Achilles, and patellar tendons [7, 13–15]. Furthermore, in this study, SST thickness was approximately 6 mm in the older subjects. Although this value was in line with that of healthy control subjects in the study of Michener et al. [7], this seemed to be attributed to similar subjects’ age and different ultrasound techniques for measuring SST thickness. Whereas our older subjects were in their 40s or older, and the average age of the control subjects was 45-year-olds in this previous study by Michener et al.; thus, the results of their control subjects in the previous study might include age-related changes in SST thickness. Additionally, whereas Michener et al. obtained short-axis images at the just anterior to the anterior-lateral margin of the acromion to measure SST thickness, we measured SST thickness on long-axis images of SST at the footprint-cartilage junction on the humeral head where it might be more distal than that in the study by Michener et al. However, SST thickness in the present study met one of the diagnostic criteria for supraspinatus tendinopathy described by Arend et al. [15] who used similar ultrasound technique as we did. Due to the absence of shoulder symptoms in our subjects, this diagnostic criterion may need to be revised; however, the current result would imply that the healthy older subjects had asymptomatic age-related SST abnormalities.

Despite our expectations, AHD was significantly greater in the older subjects, with or without arm elevation, than in the younger subjects. Greater AHD was also observed in different studies in collegiate baseball players with increased SST thickness [35]. Furthermore, an observational study reported a positive correlation between SST thickness and AHD with the arm at rest [36]. Unfortunately, the present study found no significant relationships between SST thickness and AHD. However, the difference in AHD between the younger and the older subjects across all three arm positions was as much as the difference in SST thickness between the two age groups (approximately 1 mm). Thus, a reasonable explanation for this result is that AHD enlargement in older subjects may be a positive adaptation to protect thickened supraspinatus tendons from excessive compression within the subacromial space. However, AHD possibly changes with other factors, including joint laxity [24] and muscle dysfunction [25]. Although our participants had no recent history of shoulder injuries, we should have measured the shoulder range of motion, as well as the muscle strength. In the future, comprehensive studies with subjects of a diverse range of age groups are needed to clarify age-related changes in AHD.

AHD has been measured in patients with SAI, as well as in patients with rotator cuff tears, but limited data exist regarding the age-related changes in AHD. Contrary to the present study, Hufeland et al. found no difference in AHD between the different age groups [37]. One cause of the discrepancy is the difference in methods utilized. This study used US imaging, but Hufeland et al. used magnetic resonance imaging and roentgenography. Another explanation for this discrepancy was the difference in the inclusion criteria of the younger subjects. All younger subjects were in their 20s in this study, whereas the study by Hufeland et al. included subjects ranging in age from 21 to 40 years in their younger subjects group. According to a study by Yu et al., rotator cuff tendon thickness increased with age [9]. Therefore, the younger subjects in the study by Hufeland et al. may have age-related rotator cuff tendon thickening. Because the current study suggested that enlarged AHD in older subjects may be a positive adaptation to protect the thickened supraspinatus tendon against SAI, age-related changes in rotator cuff tendon thickness could have influenced the results of AHD in the study by Hufeland et al. Thus, the current study would have more appropriately clarified the effect of aging on AHD.

This study also attempted to find age-related differences in ∆AHD and OcAHD; however, there was no significant difference between the younger and the older subjects in terms of those two measurements. In contrast to the current findings in our healthy older subjects, patients with SAI showed not only a marked reduction of AHD with arm elevation (i.e., ∆AHD) [22], but also greater OcAHD [7]. Therefore, our results suggested that the older subjects did not show SAI characteristics which have been reported in previous studies. In addition to these findings in older subjects, the current study found no significant relationships between SST thickness and AHD, as well as SST thickness and ∆AHD. As we have mentioned, the older subjects had thicker SST, presumably due to age-related tendon abnormalities; thus, our results suggest that age-related SST abnormalities occurred without SAI.

Up to now, little is known about the underlying mechanisms of age-related rotator cuff abnormalities. The underlying mechanism for rotator cuff abnormalities has been thought to include intrinsic and extrinsic factors. Whereas SAI has long been accepted as the major extrinsic factor for rotator cuff tendinopathy [20], some studies have demonstrated that surgical decompression has no beneficial effects in patients with SAI in the short-term period compared with exercise therapy or placebo surgery for patients with SAI [38–46]. Regarding long-term outcome, Ketola et al. reported no long-term beneficial effect of subacromial decompression on pain compared with placebo and exercise in the treatment of patients with rotator cuff tendinopathy at least 10 years after the treatment [47]. On the other hand, Farfaras et al. investigated clinical outcomes using functional questionnaires (the Constant score, the 36-Item Shor Form Health Survey questionnaire, and the Watson and Sonnabend score) and revealed a better long-term (a minimum of 10 years) clinical outcome with this surgical procedure than with exercise therapy alone [48]. Considering these studies, it seems likely that the long-term outcome of subacromial decompression is inconsistent, and not much is known about the long-term benefit of this surgical technique. Therefore, an unanswered question would remain as to whether SAI affects rotator cuff abnormalities in the long-term period as opposed to the short-term period. Thus, as age-related rotator cuff abnormalities seem to develop over a longer time period, we hypothesized that older individuals would display SAI characteristics when compared with younger individuals. Contrary to our hypothesis, however, our results suggested that even healthy older adults without any shoulder symptoms had age-related asymptomatic rotator cuff abnormalities without SAI. Thus, intrinsic factors (tendon blood circulation, biology, mechanical properties, morphology, and genetics [20]) may contribute to the age-related rotator cuff abnormalities rather than extrinsic factors like SAI. Indeed, morphological changes in tendons are included for intrinsic factors of tendons [20]. Consistent with the implication of this study, Sano et al. [49], as well as Factor and Dale [50], also considered that intrinsic factors were more important than extrinsic factors during the development of rotator cuff abnormalities (e.g., tendinopathy and tendon tear). Thus, any interventions that affect the intrinsic factors of the tendon would be effective in preventing age-related rotator cuff abnormalities. Unfortunately, few studies have investigated the effects of any interventions on those intrinsic factors in rotator cuff tendons. On the other hand, several researchers investigated the effects of exercises on intrinsic factors in the other tendons [51–53]. For example, Kubo et al. reported tendon mechanical properties showed different changes between isometric and plyometric training [51]. Also, low-load eccentric training for Achilles tendons increased tendon blood circulation and improved tendon collagen organization [53]. Considering these studies, various kinds of exercises may affect the intrinsic factor of tendons. Hence, further studies are required to clarify the specific type of exercise which improves the intrinsic factors of rotator cuff tendons.

It is plausible that some limitations could have influenced the results obtained. First, the characteristics of the subjects may affect the current results. The recruitment strategies of the subjects and the recruitment costs led to differences in the number of subjects between the groups in this study. However, the post hoc power analysis demonstrated sufficient statistical power. Moreover, because sex ratio differed between the groups, the sex difference may have affected the results of this study. However, sex differences are unlikely to affect the prevalence of rotator cuff tears [29]. Additionally, in our subjects, there were no differences in any variables between males and females (results are not shown). Therefore, it seems that the sex ratio does not have a major impact on the current main results. Second, while we measured AHD during arm elevation, the same amount of weight was applied in both groups. Considering that muscle strength generally decreases with aging [54, 55], the relative load in our older subjects may be greater than that in the younger subjects. A study demonstrated that the loaded condition remarkably reduced AHD compared to the unloaded condition during arm elevation [33]. Based on this effect of loading conditions on AHD, older subjects with greater relative load may have exhibited greater reduction in AHD than younger subjects with lesser relative load during arm elevation. However, this study identified greater AHD in older subjects, and also identified no significant difference in ∆AHD between different age groups. Thus, the amount of load would have hardly influenced the current results. Third, differences in anthropometric data such as arm length and scapular morphology might also affect the results of AHD. Although we did not measure arm length in our subjects, previous studies reported that height was associated with arm length and arm span [56, 57]. Given this previous study and a significant height difference between the older and younger groups, arm length may vary between groups. However, in an additional analysis of Pearson’s correlation coefficient, there was no relationship between height and AHD in elevation (*r* = − 0.256, *P* = 0.089 at 30° elevation, and *r* = − 0.220, *P* = 0.146 at 60° elevation). Therefore, the difference in arm length between older and younger subjects has little impact on the current results of AHD. By investigating the relationship between body weight/length and SST thickness, future studies will clarify the effect of anthropometric characteristics on tendon morphology. In addition, some studies reported relationships between scapular morphology and rotator cuff tendon injuries [58, 59]. Unfortunately, we did not measure the scapular morphology; thus, changes in scapular morphologies might influence the current results. Further research is needed to elucidate the causal relationship between scapular morphology and age-related rotator cuff abnormalities. Fourth, given that AHD decreases with shoulder elevation [60, 61], measuring AHD above 60° of elevation may reveal differences in AHD, ΔAHD, and OcAHD between the younger and older subjects. However, the SST insertion site passes beneath the acromion at approximately 70° of elevation [62]. Additionally, the impingement of the SST within the subacromial space was remarkable at 60° of elevation [63]. Thus, our results appear to have important implications on the effects of SAI on age-related rotator cuff abnormalities. Fifth, same as previous studies [9, 24], SST thickness measured at rest was used to calculate OcAHD in the current study. However, SST thickness may change with shoulder elevation. Therefore, it is difficult to fully understand how much compression force is exerted on SST during shoulder elevation. This point should be considered in future studies. Lastly, given that patients with various types of tendinopathies show thicker tendons, we measured SST thickness to evaluate tendon abnormalities; however, there is no evidence of the relationship between tendon thickness and histological tendon degeneration. Hence, further studies are needed on age-related changes in intrinsic factors of tendons, such as structure and mechanical properties.

## Conclusions

Whereas the older subjects showed significantly increased SST thickness and AHD compared to the younger subjects, there were no differences between the younger and the older subjects in SAI characteristics such as ∆AHD and OcAHD. These results suggest that older adults showed age-related rotator cuff abnormalities without SAI. Therefore, intrinsic factors seem to be the key pathogenesis of age-related rotator cuff degeneration, although the magnitude of the difference in SST thickness is very small and unclear in terms of clinical significance.

## Data Availability

All data generated or analyzed during this study are included in this published article.

## Abbreviations

SAI: Subacromial impingement
SST: Supraspinatus tendon
AHD: Acromiohumeral distance
∆AHD: Rate of changes in acromiohumeral distance
OcAHD: Occupation ratio
